# Evaluation of Reference Genes for Quantitative Real-Time PCR Analysis of the Gene Expression in Laticifers on the Basis of Latex Flow in Rubber Tree (*Hevea brasiliensis* Muell. Arg.)

**DOI:** 10.3389/fpls.2016.01149

**Published:** 2016-07-29

**Authors:** Jinquan Chao, Shuguang Yang, Yueyi Chen, Wei-Min Tian

**Affiliations:** Ministry of Agriculture Key Laboratory of Biology and Genetic Resources of Rubber Tree/State Key Laboratory Breeding Base of Cultivation and Physiology for Tropical Crops, Rubber Research Institute, Chinese Academy of Tropical Agricultural SciencesDanzhou, China

**Keywords:** *Hevea brasiliensis* Muell. Arg., latex flow, reference gene, qRT-PCR, geNorm software, NormFinder software

## Abstract

Latex exploitation-caused latex flow is effective in enhancing latex regeneration in laticifer cells of rubber tree. It should be suitable for screening appropriate reference gene for analysis of the expression of latex regeneration-related genes by quantitative real-time PCR (qRT-PCR). In the present study, the expression stability of 23 candidate reference genes was evaluated on the basis of latex flow by using geNorm and NormFinder algorithms. *Ubiquitin-protein ligase 2a* (*UBC2a*) and *ubiquitin-protein ligase 2b* (*UBC2b*) were the two most stable genes among the selected candidate references in rubber tree clones with differential duration of latex flow. The two genes were also high-ranked in previous reference gene screening across different tissues and experimental conditions. By contrast, the transcripts of latex regeneration-related genes fluctuated significantly during latex flow. The results suggest that screening reference gene during latex flow should be an efficient and effective clue for selection of reference genes in qRT-PCR.

## Introduction

Gene expression analysis provides the potential to explore biological processes. Northern blotting, transcriptome sequencing and qRT-PCR are universal methods that provide reliable and accurate gene expression analysis (Kubista et al., 2006; Josefsen and Nielsen, 2011; Lang et al., 2014; Le et al., 2014). Owing to its technical ease, cost-effectiveness, high specificity and accuracy, qRT-PCR has become the preferred method for detecting and measuring gene expression across different samples (Garson et al., 2005; Kubista et al., 2006). The accuracy of qRT-PCR is influenced by several factors such as the RNA extraction yield, reverse transcription and the efficiency of amplification (Huggett et al., 2005). It is necessary to normalize the expression data of the target genes with gene expression data of reference genes (Vandesompele et al., 2002; Huggett et al., 2005). A reference gene is defined as having a stable expression pattern across different tissues and experimental conditions. Generally, several housekeeping genes such as *actin* (*ACT*), *eukaryotic elongation factor 1-alpha* (*eEF-1a*), *18S rRNA, glyceraldehyde-3-phosphate dehydrogenase* (*GAPDH*) and *polyubiquitin* (*UBQ*), have been widely used as reference genes for qRT-PCR in the past decade (Bustin, 2000; Goidin et al., 2001; Kim et al., 2003; Jain et al., 2006). However, accumulating data have shown that the expression levels of these genes are variable across different developmental stages and experimental conditions (Garson et al., 2005). The use of non-validated reference genes could lead to inaccurate results and consequently, erroneous conclusions (Tricarico et al., 2002; Huggett et al., 2005; Remans et al., 2014). The 11 golden rules and Minimum Information for Publication of Quantitative Real-Time PCR Experiments (MIQE) guidelines are recommended for qRT-PCR performance in the last decade (Udvardi et al., 2008; Bustin et al., 2009, 2013).

Several statistical algorithms, such as geNorm and NormFinder, have been developed for the selection of reference genes for qRT-PCR analysis (Vandesompele et al., 2002; Andersen et al., 2004). The geNorm is a Visual Basic application tool which calculates the expression stability value (M) for all reference genes. The gene with a lower M value suggests its expression is more stable. Additionally, the pairwise variation (Vn/Vn+1) of references can also be estimated by using geNorm. It is recommended that no additional genes are required for normalization when the value of Vn/Vn+1 is below 0.15 (Vandesompele et al., 2002). As another statistical algorithm, the NormFinder ranks the stability of candidate reference genes and calculates not only the candidate reference gene variation but also the variation between subgroup samples in a sample set (Andersen et al., 2004). The lowest variety value indicates the most stable. Application of both algorithms contributes to identify the best stable reference genes for different experimental samples.

The rubber tree (*Hevea brasiliensis*) is the main source of natural rubber due to the good yield and excellent physical properties of its rubber products (Eschbach and Lacrotte, 1989). Natural rubber biosynthesis takes place in latex, the cytoplasm of highly specialized laticifer cells (Chow et al., 2012; Wang et al., 2013, 2015). In natural rubber production, latex is exploited by successive tappings. Duration of latex flow after tapping is one of crucial factors that determines rubber yield of rubber tree, and easily influenced by multiple factors such as genotypes, age of trees, seasons, etc. (Sethuraj, 1992; Raj et al., 2005; Chao et al., 2015a). Tapping causes rubber tree to be wounded and loose latex (Tang et al., 2010). The loss of latex in turn enhances latex regeneration in laticifer cells (Jacob et al., 1989). Previous studies show that tapping activates the expression of mass of latex metabolism-related genes at transcriptional level, which mainly relay on the polymerase II transcriptional complex that are responsible for eukaryotic mRNA transcription (Hahn, 2004; Hager et al., 2009; Chao et al., 2015a). Such tapping effects should be mainly ascribed to latex flow which results in latex loss from laticifer cells. To screen reference genes on the basis of latex flow may be a more efficient clue than the previously reported methods on the basis of different treatments (Li et al., 2011; Pirrello et al., 2014; Long et al., 2015). In this study, 23 candidate reference genes were evaluated for their expression stability across 18 samples obtained from three stages of latex flow of two rubber tree genotypes with differential duration of latex flow by using geNorm and NormFinder algorithms.

## Materials and Methods

### Plant Materials

In this study, we used 11-year-old regularly tapped rubber tree clones CATAS7-33-97 and CATAS8-79 with the same circumference. The trees were grown at the Experimental Station of the Rubber Research Institute of the Chinese Academy of Tropical Agricultural Sciences in Danzhou city, Hainan province. After tapping, the latex samples were respectively collected at 1, 30, and 60 min for CATAS7-33-97, and at 1, 80, and 150 min for CATAS8-79, which represented the early, middle, and late stage of latex flow, respectively. Each batch of latex samples were individual collected from three trees for each clone, and stored at -80°C until total RNA extraction.

### RNA Isolation and cDNA Synthesis

Total RNA was isolated by using the RNAprep Pure Plant Kit (Tiangen, China) and genomic DNA was eliminated according to the manufacturer’s instructions. The concentration and quality of RNA were examined using a NanoDrop 2000 (Thermo Scientific Inc., USA). The RNA integrity of the samples was checked using 1.5% agarose gel electrophoresis. Synthesis of cDNA was performed using a RevertAid TMFirst Strand cDNA Synthesis Kit (Fermentas, Canada) following the manufacturer’s protocol.

### qRT-PCR Analysis

A total of 23 housekeeping genes (*18S rRNA, ACT, ACT7a, ACT7b, ADF, ADF4, CYP2, eIF2, eIF3, eIF1Aa, eIF1Ab, FP, PTP, RH2a, RH2b, RH8, ROC3, UBC1, UBC2a, UBC2b, UBC3, UBC4*, and *YLS8*), which were evaluated in previous studies (Li et al., 2011; Chao et al., 2015b; Long et al., 2015), were chosen as candidate reference genes in the present study (Supplementary Table S1). Reactions were performed using a SYBR PrimeScript RT-PCR Kit (TaKaRa Biotechnology, Japan) on the CFX96 System (Bio-Rad Laboratories Inc., USA) as follow: 95°C for 3 min followed by 45 cycles at 95°C for 15 s, 56°C for 60 s and 72°C for 30 s, and a melt curve from 55 to 95°C increasing by 0.5°C every 30 s. Each real-time PCR reaction was performed in triplicate. The Bio-Rad CFX96 Manager 3.0 software was used for visualizing and analyzing the data, including the cycle threshold (Ct) values, amplification efficiencies (E) and melting curve of reference genes (Supplementary Table S1; **Supplementary Figure S2**). The primer efficiency of each gene was evaluated using a standard curve generated by qRT-PCR using a fivefold dilution series of cDNAs (Supplementary Table S1).

### Data Analysis

Based on the Ct values for the 23 candidate reference genes, a boxplot was drawn using GraphPad Prism 5 software^1^ to show the expression variation of each gene. The expression stability of the reference genes was analyzed using the geNorm (version 3.5^2^) and NormFinder (version 0.953^3^) software packages. To evaluate the gene expression stability, the Ct values of the candidate reference genes were converted into relative quantities using the formula E^-ΔCt^ (ΔCt = Ct value of each sample-the minimum Ct value) (Li et al., 2016), and imported into the software according to the corresponding manuals of the Norm-Finder and geNorm algorithms (Vandesompele et al., 2002; Andersen et al., 2004).

### The Expression of Latex Regeneration-Related Genes during Latex Flow

The *HbHMGR1* (Genebank accession number: AB294692) and *small rubber particle protein* (*HbSRPP*, Genebank accession number: AEH05971) were rubber biosynthesis-related genes (Sando et al., 2008; Guo et al., 2014). *HbRpb11* (Genebank accession number: KU301750) and *HbTFIIB* (Genebank accession number: KU301749) were the genes homologous to RNA polymerase II transcriptional complex members. They were selected to represent latex regeneration-related genes. Specific primers were designed using Primer Premier 5 software^4^ (Supplementary Table S1) and the amplified efficiency was checked (**Supplementary Figure S1**). The *UBC2b, 18S rRNA* and *FP* were respectively used as reference genes to normalize the transcripts of the target genes. The relative expression level of all of the target genes was evaluated using the 2^-ΔΔCt^ method (Cheng et al., 2015; Hu et al., 2016).

### Statistical Analysis

Statistical analysis was performed with SPSS Statistics 17.0^5^ by analysis of variance (ANOVA) based on Duncan’s test (multiple group comparisons) or a t-test (two group comparisons). For Duncan’s test, the capital letter or lowercase represented *p* < 0.01 or *p* < 0.05, and the same letter indicated no significant difference among groups. For *t*-test, ^∗∗∗^represented *p* < 0.001.

## Results

### Determining the Time Intervals for Latex Collection Based on the Different Durations of Latex Flow in the CATAS8-79 and CATAS7-33-97

**FIGURE 1:**
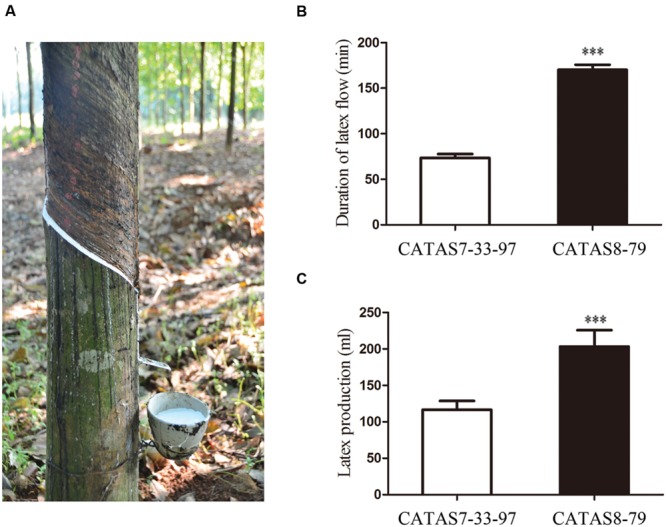
**Latex flow of rubber tree **(A)** and the difference in latex characters **(B,C)** between CATAS7-33-97 and CATAS8-79. (A)** Latex flow of rubber tree after tapping. **(B)** The difference in duration of latex flow between CATAS7-33-97 and CATAS8-79. **(C)** The difference in latex production between CATAS7-33-97 and CATAS8-79. Significant difference was indicated by the asterisks above the bars (^∗∗∗^*p* < 0.001).

Latex is usually obtained by tapping the trunk bark of rubber tree every 2-day interval (**Figure 1A**). The rubber tree clones CATAS8-79 and CATAS7-33-97 exhibited a huge difference in the duration of latex flow and latex production (**Figures 1B,C**). For CATAS8-79, the latex flow lasted for more than 160 min after tapping, which was significantly longer than that of CATAS7-33-97 (∼70 min) (**Figure 1B**). As a result, the latex production of CATAS8-79 was notably higher than that of CATAS7-33-97 (**Figure 1C**). Based on the differential duration of latex flow between the two clones, latex samples were collected at 1, 30, and 60 min after tapping for CATAS7-33-97 and at 1, 80, and 150 min after tapping for CATAS8-79, which represented the early, middle and late stages of latex flow, respectively.

### Experiment Set Classification and Transcript Abundance Evaluation

The qRT-PCR data from 18 samples were divided into three experiment sets to assess the influence of latex flow on the expression of the candidate reference genes. The first set consisted of nine samples from CATAS7-33-97. The second set was comprised of nine samples from CATAS8-79. The third set included both the first and second sample sets.

**FIGURE 2:**
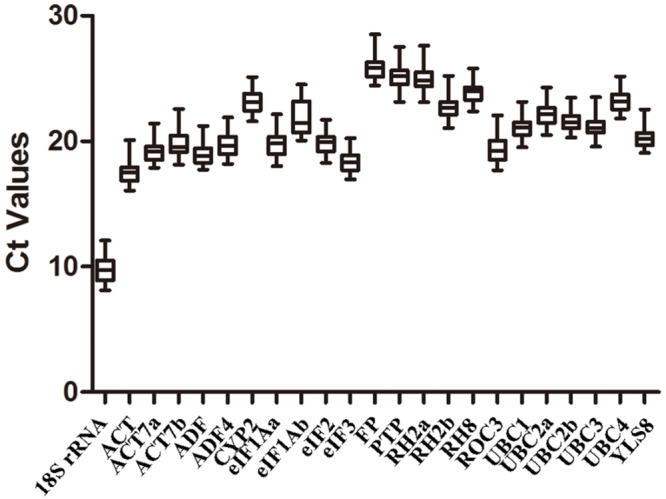
**Average cycle threshold (Ct) values for the 23 candidate reference genes upon duration of latex flow**.

The Ct values for 23 candidate reference genes were determined across all of the experimental samples using qRT-PCR (Supplementary Table S1; **Supplementary Figure S1**). The boxplot analysis was performed using GraphPad Prism 5 software (**Figure 2**). The results indicated that the selected candidate reference genes displayed a wide range of Ct values, ranging from 9 to 26. The Ct values of the majority of the candidate reference genes ranged from 19 to 22. The *18S rRNA* gene was the most abundant reference gene with the lowest mean Ct value (9.85), whereas *FP* was the least abundant reference gene with the highest mean Ct value (25.94) (**Figure 2**).

### Evaluation of Expression Stability Using geNorm

**FIGURE 3:**
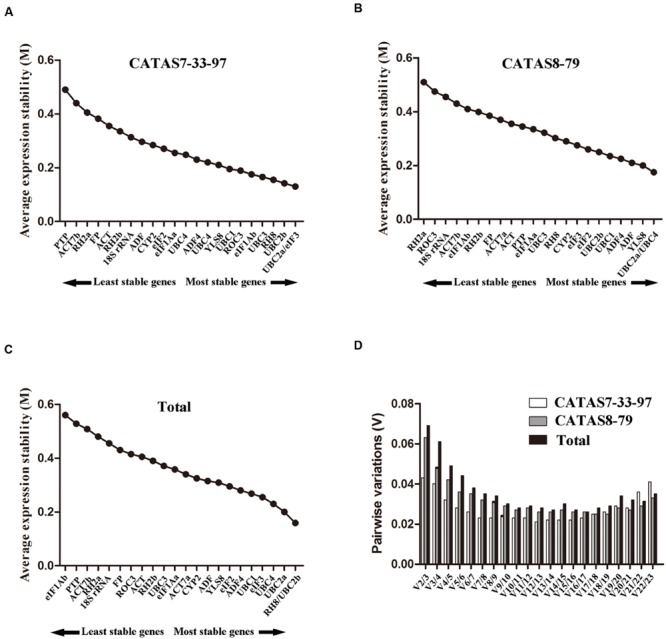
**Average gene expression stability (M) and pairwise variation (V) analyses of the candidate reference genes in different sample sets generated by the geNorm software**. The ranking of the gene expression stability performed in CATAS7-33-97 **(A)**, CATAS8-79 **(B)** and total sample **(C)**. The most stable genes are on the right, and the least stable genes are on the left. **(D)** Pairwise variation calculated to determine the minimum number of reference genes for accurate normalization.

geNorm was used to assess the expression stability (*M*) of each reference gene based on the average pair-wise variation among all of the tested genes (Vandesompele et al., 2002). The lowest *M* value indicates the most stable expression, whereas a higher *M* value is indicative of less stable expression. All of the 23 reference genes had an *M* value below the geNorm threshold of 1.5 and were ranked according to their *M* values in three sample sets (**Figures 3A–C**). For CATAS7-33-97 sample set, *UBC2a* and *eIF3* were the most stable genes with the M values of 0.13 (**Figure 3A**). The seven top-ranked genes were *UBC2a*/*eIF3, UBC2b, RH8, UBC3, eIF1Ab* and *ROC3*. For CATAS8-79 sample set, *UBC2a* and *UBC4* were the most stable genes with the *M* values of 0.17 (**Figure 3B**). The seven top-ranked genes were *UBC2a*/*UBC4, YLS8, ADF, ADF4, UBC1* and *UBC2b*. For total experiment set, *RH8* and *UBC2b* were the most stable genes with the *M* values of 0.16 (**Figure 3C**). The seven top-ranked genes were *RH8*/*UBC2b, UBC2a, UBC4, eIF3, UBC1* and *ADF4*.

To determine the optimal number of reference genes required for normalization, pairwise variation analysis (Vn/Vn + 1) was performed using the geNorm software (**Figure 3D**). The cut-off threshold value for Vn/n + 1 is 0.15 and no additional reference gene is required for normalization when the value below this value (Vandesompele et al., 2002). In the present study, the value of V2/3 was below 0.15 under all experimental sets (**Figure 3D**), which suggests that two reference genes would be sufficient for gene normalization in each experimental set.

### Evaluation of Expression Stability Using NormFinder

NormFinder is an algorithm tool to identify optimally stable reference genes by determining the expression stability (Andersen et al., 2004). Here, we use NormFinder software to further confirm the results obtained using the geNorm program. The data are listed in **Table 1**. The seven top-ranked genes were *UBC2b, RH8, eIF3, UBC3, UBC2a, eIFAa*, and *ACT7a* in the CATAS7-33-97 experiment set (**Table 1**), six of which were also present in the top seven genes in the geNorm evaluation (**Figure 3A**). The seven top-ranked genes were *UBC2a, UBC2b, UBC4, ADF4, YLS8, ADF*, and *RH8* in the CATAS8-79 experiment set (**Table 1**), six of which were also present in the top seven genes in the geNorm evaluation (**Figure 3B**). The *UBC2a, UBC2b*, and *RH8* were common between the top seven genes in both experiment sets using both NormFinder and geNorm. Moreover, they were the top three genes in the combined experiment set using both NormFinder (**Table 1**) and geNorm (**Figure 3C**).

**Table 1:** Expression stability of 23 candidate reference genes as calculated by NormFinder.

Rank	CATAS7-33-97		CATAS8-79		Total	
	Genes	Stability	Genes	Stability	Genes	Stability
1	*UBC2b*	0.032	*UBC2a*	0.059	*UBC2b*	0.091
2	*RH8*	0.096	*UBC2b*	0.097	*UBC2a*	0.096
3	*eIf3*	0.101	*UBC4*	0.109	*RH8*	0.156
4	*UBC3*	0.107	*ADF4*	0.136	*eIf3*	0.160
5	*UBC2a*	0.115	*YLS8*	0.136	*UBC1*	0.169
6	*eIF1Aa*	0.127	*ADF*	0.155	*ADF4*	0.171
7	*ACT7a*	0.133	*RH8*	0.159	*UBC4*	0.171
8	*eIF1Ab*	0.152	*UBC1*	0.162	*ADF*	0.200
9	*ROC3*	0.169	*PTP*	0.188	*ACT7a*	0.222
10	*UBC1*	0.181	*eIf3*	0.199	*eIF1Aa*	0.223
11	*YLS8*	0.185	*CYP2*	0.204	*CYP2*	0.236
12	*ADF4*	0.193	*eIF2*	0.224	*eIF2*	0.241
13	*UBC4*	0.227	*ACT*	0.232	*YLS8*	0.247
14	*ADF*	0.228	*eIF1Aa*	0.266	*ACT*	0.250
15	*RH2b*	0.236	*FP*	0.275	*RH2b*	0.260
16	*CYP2*	0.253	*UBC3*	0.276	*FP*	0.317
17	*eIF2*	0.274	*RH2b*	0.280	*UBC3*	0.317
18	*18S rRNA*	0.280	*ACT7a*	0.296	*ROC3*	0.334
19	*ACT*	0.281	*eIF1Ab*	0.339	*18S rRNA*	0.366
20	*FP*	0.374	*ACT7b*	0.371	*RH2a*	0.446
21	*RH2a*	0.381	*18S rRNA*	0.388	*PTP*	0.464
22	*ACT7b*	0.558	*ROC3*	0.447	*ACT7b*	0.471
23	*PTP*	0.649	*RH2a*	0.517	*eIF1Ab*	0.560

### Influence of Latex Flow on the Expression of Latex Regeneration-Related Genes

**FIGURE 4:**
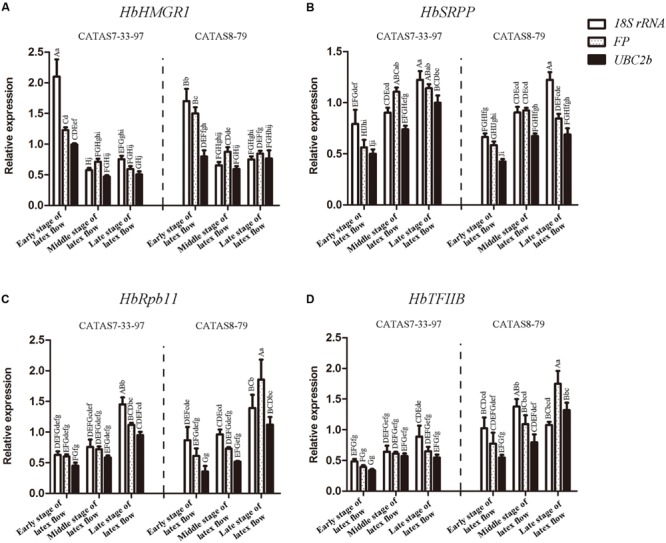
**Relative expression of *HbHMGR1***(A)**, *HbSRPP***(B)**, *HbRpb11***(C)** and *HbTFIIB***(D)** by using *18S rRNA, FP* or *UBC2b* for normalization.** Error bars for qRT-PCR showed the standard deviation of three replicates. Different letters indicate statistically significant difference in relative expression of each gene among different tissues. The capital letter represents *p* < 0.01 while lowercase represents *p* < 0.05. Analyses were performed with SPSS Statistics 17.0. Bars indicate the standard deviation of three biological replicates in triplicate.

The *HbHMGR1, HbSRPP1, HbTFII*B and *HbRpb11* were selected to analyze the expression of latex regeneration-related gene at different stages of latex flow. The relative expression was respectively normalized against reference genes *UBC2b, 18S rRNA* and *FP*. The *UBC2b* was the most stable reference gene while the traditionally used *18S rRNA* and the more stable *FP* reported by Li et al. (2011) were less stable in the present study. The transcript level of all the four genes were significantly fluctuated during latex flow when normalized by any of the three reference genes (**Figure 4**). The fluctuating pattern of each target gene was, however, different to some extent among the three normalizations (**Figure 4**). There was no difference in the transcript level of *HbHMGR1* between “middle” and “late” stages of latex flow by using *UBC2b* normalization in either CATAS7-33-97 or CATAS8-79 (**Figure 4A**). But it showed significant difference (*p* < 0.01) by using *18S rRNA* normalization in CATAS7-33-97 and difference (*p* < 0.05) by using *FP* normalization in CATAS8-79 (**Figure 4A**). The transcript level of *HbSRPP* between “middle” and “late” stages showed significant difference (*p* < 0.01) in CATAS7-33-97 while had no difference in CATAS8-79 by using *UBC2b* normalization (**Figure 4B**). It had no difference by using *FP* normalization while showed significant difference (*p* < 0.01) by using *18S rRNA* in either CATAS7-33-97 or CATAS8-79 (**Figure 4B**). The relative transcript level of *HbRpb11* had no difference between “middle” and “late” stages of latex flow in CATAS7-33-97 by using either *UBC2b* or *FP* normalization while significant difference (*p* < 0.01) by using *18S rRNA* normalization (**Figure 4C**). It showed difference in CATAS8-79 for *18S rRNA* normalization and significant difference for both *UBC2b* and *FP* normalization (**Figure 4C**) The *UBC2b* normalized expression pattern of *HbTFIIB* in either CATAS7-33-97 or CATAS8-79 was similar with the *FP* normalized but different from the *18S rRNA* normalized expression pattern (**Figure 4D**).

## Discussion

Accurate interpretation of the qRT-PCR results mainly depends on the use of stable reference genes for data normalization to minimize non-biological variations among samples (Huggett et al., 2005). However, increasing evidence shows that the best reference gene significantly depends on the experimental conditions (Garson et al., 2005). Therefore, validating suitable reference genes is necessary to obtain accurate gene expression patterns under specific conditions using qRT-PCR (Ji et al., 2014; Wang et al., 2014; Delporte et al., 2015; Kong et al., 2015; Niu et al., 2015).

The rubber tree is one of the most economically important members of the genus *Hevea* because the milky latex exploited from the tree is the primary source of natural rubber. The latex exploitation is an effective factor that enhances latex regeneration (Jacob et al., 1989). After tapping, the loss of latex from latex flow results in significant changes in turgor pressure of laticifer cells, which may significantly reprogram the transcriptional regulation of latex regeneration-related genes in laticifer cells at the molecular level (Chao et al., 2015a). In the present study, we showed that not only the rubber biosynthesis-related genes such as *HbHMGR1* and *HbSRPP* significantly fluctuated, but also the primary metabolism-related genes such as *HbTFIIB* and *HbRpb11*, the homologs of transcriptional complex members, were notably reprogrammed during latex flow (**Figure 4**). The results indicate that latex regeneration-related genes are strongly influenced in the process of latex flow. This factor is largely ignored when other factors such as tapping and hormone applications are considered in screening reference genes (Li et al., 2011; Pirrello et al., 2014; Long et al., 2015).

**FIGURE 5:**
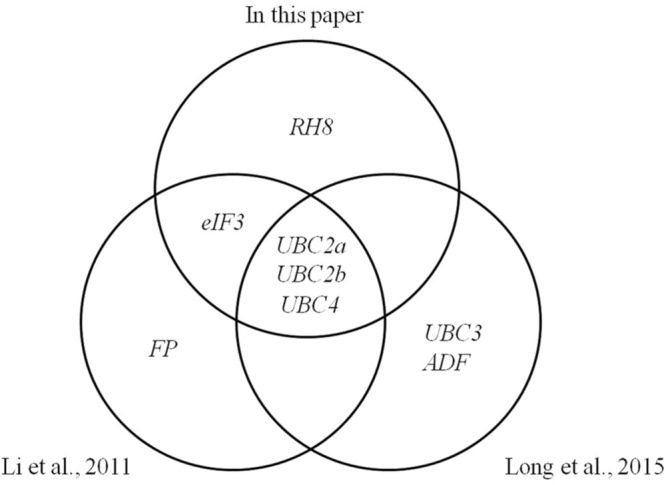
**Comparing suitable reference genes screened here with previous studies on rubber tree.** The top five best references of each experimental group were analyzed here.

The expression stability of 22 candidate reference genes was evaluated based on tapping, genotypes, tissues, and plant hormones (Li et al., 2011). The authors suggested that the top five reference genes were *UBC2a, UBC2b, UBC4, eIF3*, and *FP*. Thereafter, 20 of the 22 candidate reference genes were evaluated using successive tapping and the top five reference genes were *UBC2a, UBC2b, UBC4, UBC3*, and *ADF* (Long et al., 2015). Three genes were identical between the top five genes. The *RH2b, UBC2a, RH8* are the top three best references for abiotic and harvesting stress of rubber tree by screening 11 of the 22 candidate genes and simply evaluating based on the standard deviation and coefficient of variance of Ct value that generated by qRT-PCR (Pirrello et al., 2014). In the present study, the selected 23 candidate reference genes included the 22 candidate reference genes (Li et al., 2011) and an *ACT* gene (Chao et al., 2015b). The expression stability of all the 23 candidate reference genes was evaluated on the basis of latex flow using geNorm and NormFinder algorithms. The top five genes were *UBC2a, UBC2b, UBC4, eIF3*, and *RH8*. Four of the five genes are identical to the genes reported by Li et al. (2011) and three of the five genes are identical to those reported by Long et al. (2015). It is interesting that three *UBC* family members (*UBC2a, UBC2b*, and *UBC4*), are common among these experimental groups of rubber trees (**Figure 5**).

*UBC* is a large gene family comprising dozens of members, which widely expands in eukaryotes (Sheng et al., 2012). In the present study, two *UBCs*, the *UBC2a* and *UBC2b*, had the best stability based on latex flow in rubber trees. It is interesting that *UBC2a* and *UBC2b* are also stable in laticifer cells and bark tissue of rubber trees. For example, *UBC2b* was also found to be highly stable across multiple experimental conditions, such as hormone treatments, genotypes and latex regeneration between two tapping intervals (Li et al., 2011; Long et al., 2015). *UBC2a* was the most stable gene in the bark tissues of rubber trees treated with coronatine (data unpublished). The homolog of *UBC2a* and *UBC2b* is also stable in many species such as *Brachypodium distachyton* (Chambers et al., 2012), *Trifolium pretense* (Mehdi Khanlou and Van Bockstaele, 2012), *Lolium perenne* (Huang et al., 2014), and *Withania somnifera* (Singh et al., 2015) under different experimental conditions. In plants, *UBC2a* and *UBC2b* encode ubiquitin-conjugating enzyme subunits that are necessary for the degradation of ubiquitinated proteins by the eukaryotic conserved 26S protein degradation pathway (Stone, 2014). Protein ubiquitination is mediated by the sequential action of E1 (ubiquitin-activating enzyme, UBA), E2 (ubiquitin-conjugating enzyme, UBC), and E3 (ubiquitin-ligating enzyme) (E et al., 2015). E2 UBC enzymes accept activated ubiquitin from E1 through a transthiolation reaction, and then transfer ubiquitin to either a substrate directly aided by E3 or a cysteine of ubiquitin protein ligase through a second transthiolation reaction that then transfers ubiquitin to the substrate (Jue et al., 2015). The housekeeping of *UBCs* may attribute to the fact that UBCs function as an intermediate step of the ubiquitin-proteasome degradation system to control the abundance of cellular proteins required for plant growth and development (Stone and Callis, 2007; Vierstra, 2009). Taken together, screening reference genes on the basis of latex flow should be an efficient and effective clue for selection of reference genes in qRT-PCR. The *UBC2a* and *UBC2b* could be used as suitable reference genes for qRT-PCR analysis of latex regeneration-related genes following different treatments.

## Author Contributions

JC designed and carried out the experiment of this study, and wrote the manuscript. SY, YC participated and analyzed data in the experiment. WT planned the study and participated in the design of the experiment. All authors have read and approved the manuscript in its final form.

## Conflict of Interest Statement

The authors declare that the research was conducted in the absence of any commercial or financial relationships that could be construed as a potential conflict of interest.

## Footnotes

1 http://www.graphpad.com/

2 https://genorm.cmgg.be/

3 http://moma.dk/normfinder-software/

4 http://www.premierbiosoft.com/primerdesign/

5 http://www.spss.com.cn/

## Supplementary Material

FIGURE S1 **The melting curves of the 23 candidate reference genes and four latex regeneration-related genes**.

## Abbreviations

18S rRNA: 18s ribosomal RNA
ACT7a: Actin (ACTIN7)
ACT7b: Actin (ACTIN7)
ADF: Actin depolymerizing factor
ADF4: Actin depolymerizing factor 4
Ct: threshold cycle
eIF1Aa: Eukaryotic translation initiation factor 1A
eIF1Ab: Eukaryotic translation initiation factor 1A
eIF2: Eukaryotic translation initiation factor
eIF3: Eukaryotic translation initiation factor
FP: F-box family protein
HMGR1: 3-Hydroxy-3-methylglutaryl-coenzyme A reductase
PTP: Trosine phosphatase
qRT-PCR: Quantitative real-time PCR
RH2a: DEAD box RNA helicase, RH2
RH2b: DEAD box RNA helicase, RH2
ROC3: Cytosolic cyclophilin (ROC3)
SRPP: Small rubber particle protein
TFIIB: Transcription initiation factor IIB
UBC1: Ubiquitin-protein ligase
UBC2a: Ubiquitin-protein ligase (ATUBC2)
UBC2b: Ubiquitin-protein ligase (ATUBC2)
UBC3: Ubiquitin-protein ligase
UBC4: Ubiquitin-protein ligase
YLS8: Mitosis protein YLS8

## References

[B1] Andersen C. L. Jensen J. L. Orntoft T. F. (2004). Normalization of real-time quantitative reverse transcription-PCR data: a model-based variance estimation approach to identify genes suited for normalization, applied to bladder and colon cancer data sets. *Cancer Res.* 64 5245–5250. 10.1158/0008-5472.CAN-04-0496 15289330

[B2] Bustin S. A. (2000). Absolute quantification of mRNA using real-time reverse transcription polymerase chain reaction assays. *J. Mol. Endocrinol.* 25 169–193. 10.1677/jme.0.0250169 11013345

[B3] Bustin S. A. Benes V. Garson J. A. Hellemans J. Huggett J. Kubista M. (2009). The MIQE guidelines: minimum information for publication of quantitative real-time PCR experiments. *Clin. Chem.* 55 611–622. 10.1373/clinchem.2008.112797 19246619

[B4] Bustin S. A. Benes V. Garson J. A. Hellemans J. Huggett J. Kubista M. (2013). The need for transparency and good practices in the qPCR literature. *Nat. Methods* 10 1063–1067. 10.1038/nmeth.2697 24173381

[B5] Chambers J. P. Behpouri A. Bird A. Ng C. K. (2012). Evaluation of the use of the polyubiquitin genes, Ubi4 and Ubi10 as reference genes for expression studies in *Brachypodium distachyon*. *PLoS ONE* 7:e49372 10.1371/journal.pone.0049372 PMC3498167 23166649

[B6] Chao J. Chen Y. Wu S. Tian W. M. (2015a). Comparative transcriptome analysis of latex from rubber tree clone CATAS8-79 and PR107 reveals new cues for the regulation of latex regeneration and duration of latex flow. *BMC Plant Biol.* 15:104 10.1186/s12870-015-0488-3 PMC4410575 25928745

[B7] Chao J. Zhang S. Chen Y. Tian W. M. (2015b). Cloning, heterologous expression and characterization of ascorbate peroxidase (APX) gene in laticifer cells of rubber tree (*Hevea brasiliensis* Muell. Arg.). *Plant Physiol. Biochem.* 97 331–338. 10.1016/j.plaphy.2015.10.023 26519821

[B8] Cheng H. M. Chern Y. Chen I. H. Liu C. Li S. H. Chun S. J. (2015). Effects on murine behavior and lifespan of selectively decreasing expression of mutant huntingtin allele by supt4h knockdown. *PLoS Genet.* 11:e1005043 10.1371/journal.pgen.1005043 PMC4356588 25760041

[B9] Chow K. S. Mat-Isa M. N. Bahari A. Ghazali A. K. Alias H. Mohd-Zainuddin Z. (2012). Metabolic routes affecting rubber biosynthesis in *Hevea brasiliensis* latex. *J. Exp. Bot.* 63 1863–1871. 10.1093/jxb/err363 22162870 PMC3295384

[B10] Delporte M. Legrand G. Hilbert J. L. Gagneul D. (2015). Selection and validation of reference genes for quantitative real-time PCR analysis of gene expression in *Cichorium intybus*. *Front. Plant Sci.* 6:651 10.3389/fpls.2015.00651 PMC4539468 26347767

[B11] E Z. Zhang Y. Li T. Wang L. Zhao H. (2015). Characterization of the ubiquitin-conjugating enzyme gene family in rice and evaluation of expression profiles under abiotic stresses and hormone treatments. *PLoS ONE* 10:e0122621 10.1371/journal.pone.0122621 PMC4406754 25902049

[B12] Eschbach J. M. Lacrotte R. (1989). “Factors influencing response to hormonal yield stimulation: limits of this stimulation,” in *Physiology of Rubber Tree Latex* eds d’Auzac J. Jacob J. L. Chrestin H. (Boca Raton, FL: CRC Press) 321–342.

[B13] Garson J. A. Grant P. R. Ayliffe U. Ferns R. B. Tedder R. S. (2005). Real-time PCR quantitation of hepatitis B virus DNA using automated sample preparation and murine cytomegalovirus internal control. *J. Virol Methods* 126 207–213. 10.1016/j.jviromet.2005.03.001 15847939

[B14] Goidin D. Mamessier A. Staquet M. J. Schmitt D. Berthier-Vergnes O. (2001). Ribosomal 18S RNA prevails over glyceraldehyde-3-phosphate dehydrogenase and beta-actin genes as internal standard for quantitative comparison of mRNA levels in invasive and noninvasive human melanoma cell subpopulations. *Anal. Biochem.* 295 17–21. 10.1006/abio.2001.5171 11476540

[B15] Guo D. Li H. L. Tang X. Peng S. Q. (2014). Molecular and functional characterization of the HbSRPP promoter in response to hormones and abiotic stresses. *Transgenic Res.* 23 331–340. 10.1007/s11248-013-9753-0 24043397

[B16] Hager G. L. McNally J. G. Misteli T. (2009). Transcription dynamics. *Mol. Cell* 35 741–753. 10.1016/j.molcel.2009.09.005 19782025 PMC6326382

[B17] Hahn S. (2004). Structure and mechanism of the RNA polymerase II transcription machinery. *Nat. Struct. Mol. Biol.* 11 394–403. 10.1038/nsmb763 15114340 PMC1189732

[B18] Hu M. Hu W. Xia Z. Zhou X. Wang W. (2016). Validation of reference genes for relative quantitative gene expression studies in cassava (*Manihot esculenta* Crantz) by using quantitative real-time PCR. *Front. Plant Sci.* 7:680 10.3389/fpls.2016.00680 PMC4871855 27242878

[B19] Huang L. Yan H. Jiang X. Yin G. Zhang X. Qi X. (2014). Identification of candidate reference genes in perennial ryegrass for quantitative RT-PCR under various abiotic stress conditions. *PLoS ONE* 9:e93724 10.1371/journal.pone.0093724 PMC3974806 24699822

[B20] Huggett J. Dheda K. Bustin S. Zumla A. (2005). Real-time RT-PCR normalisation; strategies and considerations. *Genes Immun.* 6 279–284. 10.1038/sj.gene.6364190 15815687

[B21] Jacob J. L. Prevot J. C. Roussel D. Lacrotte R. Serres E. d’Auzac J. (1989). “Yield limiting factors, latex physiological parameters, latex diagnosis, and clonal typology,” in *Physiology of Rubber Tree Latex* eds d’Auzac J. Jacob J. L. Chrestin H. (Boca Raton, FL: CRC Press) 345–382.

[B22] Jain M. Nijhawan A. Tyagi A. K. Khurana J. P. (2006). Validation of housekeeping genes as internal control for studying gene expression in rice by quantitative real-time PCR. *Biochem. Biophys. Res. Commun.* 345 646–651. 10.1016/j.bbrc.2006.04.140 16690022

[B23] Ji Y. Tu P. Wang K. Gao F. Yang W. Zhu Y. (2014). Defining reference genes for quantitative real-time PCR analysis of anther development in rice. *Acta Biochim. Biophys. Sin. (Shanghai)* 46 305–312. 10.1093/abbs/gmu002 24492537

[B24] Josefsen K. Nielsen H. (2011). Northern blotting analysis. *Methods Mol. Biol.* 703 87–105. 10.1007/978-1-59745-248-9_7 21125485

[B25] Jue D. Sang X. Lu S. Dong C. Zhao Q. Chen H. (2015). Genome-wide identification, phylogenetic and expression analyses of the ubiquitin-conjugating enzyme gene family in maize. *PLoS ONE* 10:e0143488 10.1371/journal.pone.0143488 PMC4659669 26606743

[B26] Kim B. R. Nam H. Y. Kim S. U. Kim S. I. Chang Y. J. (2003). Normalization of reverse transcription quantitative-PCR with housekeeping genes in rice. *Biotechnol. Lett.* 25 1869–1872. 10.1023/A:1026298032009 14677714

[B27] Kong Q. Yuan J. Gao L. Zhao L. Cheng F. Huang Y. (2015). Evaluation of appropriate reference genes for gene expression normalization during watermelon fruit development. *PLoS ONE* 10:e0130865 10.1371/journal.pone.0130865 PMC4481515 26110539

[B28] Kubista M. Andrade J. M. Bengtsson M. Forootan A. Jonak J. Lind K. (2006). The real-time polymerase chain reaction. *Mol. Aspects Med.* 27 95–125. 10.1016/j.mam.2005.12.007 16460794

[B29] Lang V. Usadel B. Obermeyer G. (2014). De novo sequencing and analysis of the lily pollen transcriptome: an open access data source for an orphan plant species. *Plant Mol. Biol.* 87 69–80. 10.1007/s11103-014-0261-2 25341867

[B30] Le M. Q. Pagter M. Hincha D. K. (2014). Global changes in gene expression, assayed by microarray hybridization and quantitative RT-PCR, during acclimation of three *Arabidopsis thaliana* accessions to sub-zero temperatures after cold acclimation. *Plant Mol. Biol.* 87 1–15. 10.1007/s11103-014-0256-z 25311197

[B31] Li H. Qin Y. Xiao X. Tang C. (2011). Screening of valid reference genes for real-time RT-PCR data normalization in *Hevea brasiliensis* and expression validation of a sucrose transporter gene HbSUT3. *Plant Sci.* 181 132–139. 10.1016/j.plantsci.2011.04.014 21683878

[B32] Li J. Han J. Hu Y. Yang J. (2016). Selection of reference genes for quantitative real-time PCR during flower development in tree *Peony* (*Paeonia suffruticosa* Andr.). *Front. Plant Sci.* 7:516 10.3389/fpls.2016.00516 PMC4838814 27148337

[B33] Long X. He B. Gao X. Qin Y. Yang J. Fang Y. (2015). Validation of reference genes for quantitative real-time PCR during latex regeneration in rubber tree. *Gene* 563 190–195. 10.1016/j.gene.2015.03.026 25791491

[B34] Mehdi Khanlou K. Van Bockstaele E. (2012). A critique of widely used normalization software tools and an alternative method to identify reliable reference genes in red clover (*Trifolium pratense* L.). *Planta* 236 1381–1393. 10.1007/s00425-012-1682-2 22718310

[B35] Niu X. Qi J. Zhang G. Xu J. Tao A. Fang P. (2015). Selection of reliable reference genes for quantitative real-time PCR gene expression analysis in Jute (*Corchorus capsularis*) under stress treatments. *Front. Plant Sci.* 6:848 10.3389/fpls.2015.00848 PMC4604321 26528312

[B36] Pirrello J. Leclercq J. Dessailly F. Rio M. Piyatrakul P. Kuswanhadi K. (2014). Transcriptional and post-transcriptional regulation of the jasmonate signalling pathway in response to abiotic and harvesting stress in *Hevea brasiliensis*. *BMC Plant Biol.* 14:341 10.1186/s12870-014-0341-0 PMC4274682 25443311

[B37] Raj S. Das G. Pothen J. Dey S. K. (2005). Relationship between latex yield of *Hevea brasiliensis* and antecedent environmental parameters. *Int. J. Biometeorol.* 49 189–196. 10.1007/s00484-004-0222-6 15290432

[B38] Remans T. Keunen E. Bex G. J. Smeets K. Vangronsveld J. Cuypers A. (2014). Reliable gene expression analysis by reverse transcription-quantitative PCR: reporting and minimizing the uncertainty in data accuracy. *Plant Cell* 26 3829–3837. 10.1105/tpc.114.130641 25361954 PMC4247583

[B39] Sando T. Takaoka C. Mukai Y. Yamashita A. Hattori M. Ogasawara N. (2008). Cloning and characterization of mevalonate pathway genes in a natural rubber producing plant, *Hevea brasiliensis*. *Biosci. Biotechnol. Biochem.* 72 2049–2060. 10.1271/bbb.80165 18685207

[B40] Sethuraj M. R. (1992). “Yield components in *Hevea Brasiliensis*,” in *Natural Rubber: Biology, Cultivation and Technology* eds Sethuraj M. R. Mathew N. M. (Amsterdam Elsevier Press) 137–163.

[B41] Sheng Y. Hong J. H. Doherty R. Srikumar T. Shloush J. Avvakumov G. V. (2012). A human ubiquitin conjugating enzyme (E2)-HECT E3 ligase structure-function screen. *Mol. Cell Proteomics* 11 329–341. 10.1074/mcp.O111.013706 22496338 PMC3412965

[B42] Singh V. Kaul S. C. Wadhwa R. Pati P. K. (2015). Evaluation and selection of candidate reference genes for normalization of quantitative RT-PCR in *Withania somnifera* (L.) Dunal. *PLoS ONE* 10:e0118860 10.1371/journal.pone.0118860 PMC4359125 25769035

[B43] Stone S. L. (2014). The role of ubiquitin and the 26S proteasome in plant abiotic stress signaling. *Front. Plant Sci.* 5:135 10.3389/fpls.2014.00135 PMC3997020 24795732

[B44] Stone S. L. Callis J. (2007). Ubiquitin ligases mediate growth and development by promoting protein death. *Curr. Opin. Plant Biol.* 10 624–632. 10.1016/j.pbi.2007.07.010 17851112

[B45] Tang C. Huang D. Yang J. Liu S. Sakr S. Li H. (2010). The sucrose transporter HbSUT3 plays an active role in sucrose loading to laticifer and rubber productivity in exploited trees of *Hevea brasiliensis* (para rubber tree). *Plant Cell Environ.* 33 1708–1720. 10.1111/j.1365-3040.2010.02175.x 20492551

[B46] Tricarico C. Pinzani P. Bianchi S. Paglierani M. Distante V. Pazzagli M. (2002). Quantitative real-time reverse transcription polymerase chain reaction: normalization to rRNA or single housekeeping genes is inappropriate for human tissue biopsies. *Anal. Biochem.* 309 293–300. 10.1016/S0003-2697(02)00311-1 12413463

[B47] Udvardi M. K. Czechowski T. Scheible W. R. (2008). Eleven golden rules of quantitative RT-PCR. *Plant Cell* 20 1736–1737. 10.1105/tpc.108.061143 18664613 PMC2518243

[B48] Vandesompele J. De Preter K. Pattyn F. Poppe B. Van Roy N. De Paepe A. (2002). Accurate normalization of real-time quantitative RT-PCR data by geometric averaging of multiple internal control genes. *Genome Biol.* 3 1–11. 10.1186/gb-2002-3-7-research0034 PMC126239 12184808

[B49] Vierstra R. D. (2009). The ubiquitin-26S proteasome system at the nexus of plant biology. *Nat. Rev. Mol. Cell Biol.* 10 385–397. 10.1038/nrm2688 19424292

[B50] Wang H. Wang J. Jiang J. Chen S. Guan Z. Liao Y. (2014). Reference genes for normalizing transcription in diploid and tetraploid *Arabidopsis*. *Sci. Rep.* 4:6781 10.1038/srep06781 PMC4209459 25345678

[B51] Wang X. Shi M. Wang D. Chen Y. Cai F. Zhang S. (2013). Comparative proteomics of primary and secondary lutoids reveals that chitinase and glucanase play a crucial combined role in rubber particle aggregation in *Hevea brasiliensis*. *J. Proteome Res.* 12 5146–5159. 10.1021/pr400378c 23991906

[B52] Wang X. Wang D. Sun Y. Yang Q. Chang L. Wang L. (2015). Comprehensive proteomics analysis of laticifer latex reveals new insights into ethylene stimulation of natural rubber production. *Sci. Rep.* 5:13778 10.1038/srep13778 PMC4562231 26348427

