# Supplementary material for: Evaluation of Reference Genes for Quantitative Real-Time PCR Analysis of the Gene Expression in Laticifers on the Basis of Latex Flow in Rubber Tree (Hevea brasiliensis Muell. Arg.)
Source: Front Plant Sci. 2016 Jul 29;7:1149. doi: 10.3389/fpls.2016.01149 (PMC4965454; doi:10.3389/fpls.2016.01149)
Supplement: Supplementary file 1 [file Table_1.DOC]

**Additional file 1**

**Table S1.** Primers used in this paper.

|  | **Unigene ID** | **Primer sequence** | **Annotation** | **Amplicon length (bp)** | **Amplification**  **Efficiency** | **GenBank Accession** |
| --- | --- | --- | --- | --- | --- | --- |
| **Candidate reference genes** | | | | | | |
|  | *18S RNA* | Forward: GCTCGAAGACGATCAGATACC  Reverse: TTCAGCCTTGCGACCATAC | 18s ribosomal RNA | 146 | 94.3% | AB268099 |
|  | *ACT* | Forward: GATTCCGTTGCCCAGAAGTC  Reverse: CACCACTCAGCACAATGTTACC | Actin | 144 | 95% | JF270598 |
|  | *ACT7a* | Forward: GGCACTTTGGTACTCAAGTC  Reverse: GAAGCATCCCAATCACTCTC | Actin (ACTIN7) | 102 | 93.8% | HQ260074 |
|  | *ACT7b* | Forward: GTTGGGATGGGGCAGAAAG  Reverse: TGCTGACAATACCGTGCTCAA | Actin (ACTIN7) | 100 | 88.1% | HQ395755 |
|  | *ADF* | Forward: GCATATCGGCACTCATCAG  Reverse: GGAGAAGGCAAAGCAAGTC | Actin depolymerizing factor 4 (ADF4) | 99 | 100.8% | HM126477 |
|  | *ADF4* | Forward: GTGCCGATATGCTGTCTATGATTT  Reverse: TCCCTCTTGAACCTGTCCTTG | Actin depolymerizing factor 4 (ADF4) | 147 | 89.8% | HQ268020 |
|  | *CYP2* | Forward: CTGTACTGCCAAGACTGAG  Reverse: ATGGAGCTATTCGCGGATAC | Cyclophilin (CYP2) | 86 | 88.1% | HQ268021 |
|  | *eIF1Aa* | Forward: GCGTGACTATCAGGACGACAA  Reverse: CAAGACCTCCAGCAATACCCTC | Eukaryotic translation initiation factor (1A) | 128 | 92.2% | HQ268022 |
|  | *eIF1Ab* | Forward: GCGTGACTATCAGGACGACAA  Reverse: CAAGACCTCCAGCAATACCCT | Eukaryotic translation initiation factor (1A) | 135 | 99.5% | HQ268023 |
|  | *eIF2* | Forward: CGACCTTTGATCCGTTTGCT  Reverse: CTTCCTACCATTCCGTTGCT | Eukaryotic translation initiation factor | 98 | 95% | HQ268024 |
|  | *eIf3* | Forward: CTGCACAGTAGTCAAGCTCTTTC  Reverse: CGAAACCCAGATTCCTTCTACCT | Eukaryotic translation initiation factor | 133 | 95.7% | HQ268025 |
|  | *FP* | Forward: ATCAGCTTGACGCAGGTTAC  Reverse: GAAACAGACGCTTCTGAGTG | F-box family protein | 99 | 95.5% | HQ268026 |
|  | *PTP* | Forward: TGCCCTCCAATTCAACTG  Reverse: TGCACCACAACGACATTC | Trosine phosphatase | 86 | 91.5% | HQ268027 |
|  | *RH2a* | Forward: CCATCACCCAGGCTGTTATTTTC  Reverse: GTAAAGTTATTGCTCCGCATCTTC | DEAD box RNA helicase,RH2 | 82 | 86.5% | HQ323242 |
|  | *RH2b* | Forward: CGACCAAGTTTTCATTTCGGGTG  Reverse: AGTCTCTTCTTTGCTGGGGTTG | DEAD box RNA helicase,RH2 | 150 | 97.5% | HQ323243 |
|  | *RH8* | Forward: TCACAGGGTTGGTAGATCAG  Reverse: CCAAGCTCTTGCTCAATCC | DEAD box RNA helicase,RH8 | 105 | 95.6% | HQ323244 |
|  | *ROC3* | Forward: ATGGAGCTATTCGCGGATAC  Reverse: GGTGGAAACTCGATCCTTTG | Cytosolic cyclophilin (ROC3) | 121 | 84% | EU295481 |
|  | *UBC1* | Forward: ACTTTGCCCTTGATGCCT  Reverse: GCGTTAGTTGGGATCTGGT | Ubiquitin-protein ligase | 102 | 87.3% | HQ323245 |
|  | *UBC2a* | Forward: CATTTATGCGGATGGAAGCA  Reverse: CAGGGGAGTTTGGATTTGGA | Ubiquitin-protein ligase (ATUBC2) | 125 | 94.7% | HQ323246 |
|  | *UBC2b* | Forward: CGACCAAGTTTTCATTTCGGGTG  Reverse: AGTCTCTTCTTTGCTGGGGTTG | Ubiquitin-protein ligase (ATUBC2) | 95 | 94.4% | HQ323247 |
|  | *UBC3* | Forward: CCCTGATGATCCACTTTCTG  Reverse: ACCATCATGCACCACTTG | Ubiquitin-protein ligase | 113 | 88.1% | HQ323348 |
|  | *UBC4* | Forward: TCCTTATGAGGGCGGAGTC  Reverse: CAAGAACCGCACTTGAGGAG | Ubiquitin-protein ligase | 82 | 87.5% | HQ323249 |
|  | *YLS8* | Forward: CCTCGTCGTCATCCGATTC  Reverse: CAGGCACCTCAGTGATGTC | Mitosis protein YLS8 | 131 | 99.2% | HQ323250 |
| **Latex regeneration-related genes** | | | | | | |
|  | *HbHMGR1* | Forward: GCTGTTATATGAAGTATGGAGATAGG  Reverse: AAGGGTAGAGAGAGAAGTAGAGG | Hydroxymethylglutaryl-CoA reductase 1 | 105 | 95.4% | AB294692 |
|  | *HbSRPP* | Forward: GCTGGAGTTTATGCTGTAGATTC  Reverse: TTCACCACATTCTCAATAGTATCG | Small rubber particle protein | 104 | 93.2% | HQ640231 |
|  | *HbRpb11* | Forward: GGTGTGGTCCTCCCTCT  Reverse: TTTGAACTCCTCCGTGC | RNA polymerase II subunit RPB11 | 179 | 91.8% | KU301750 |
|  | *HbTFIIB* | Forward: GGACCACCAACACGAAC  Reverse: GGCTGACACCTACTGCC | RNA polymerase II subunit TFIIB | 183 | 96.3% | KU301749 |
