# Supplementary figures and images for: Evaluation of Reference Genes for Quantitative Real-Time PCR Analysis of the Gene Expression in Laticifers on the Basis of Latex Flow in Rubber Tree (Hevea brasiliensis Muell. Arg.)
Source: Front Plant Sci. 2016 Jul 29;7:1149. doi: 10.3389/fpls.2016.01149 (PMC4965454; doi:10.3389/fpls.2016.01149)

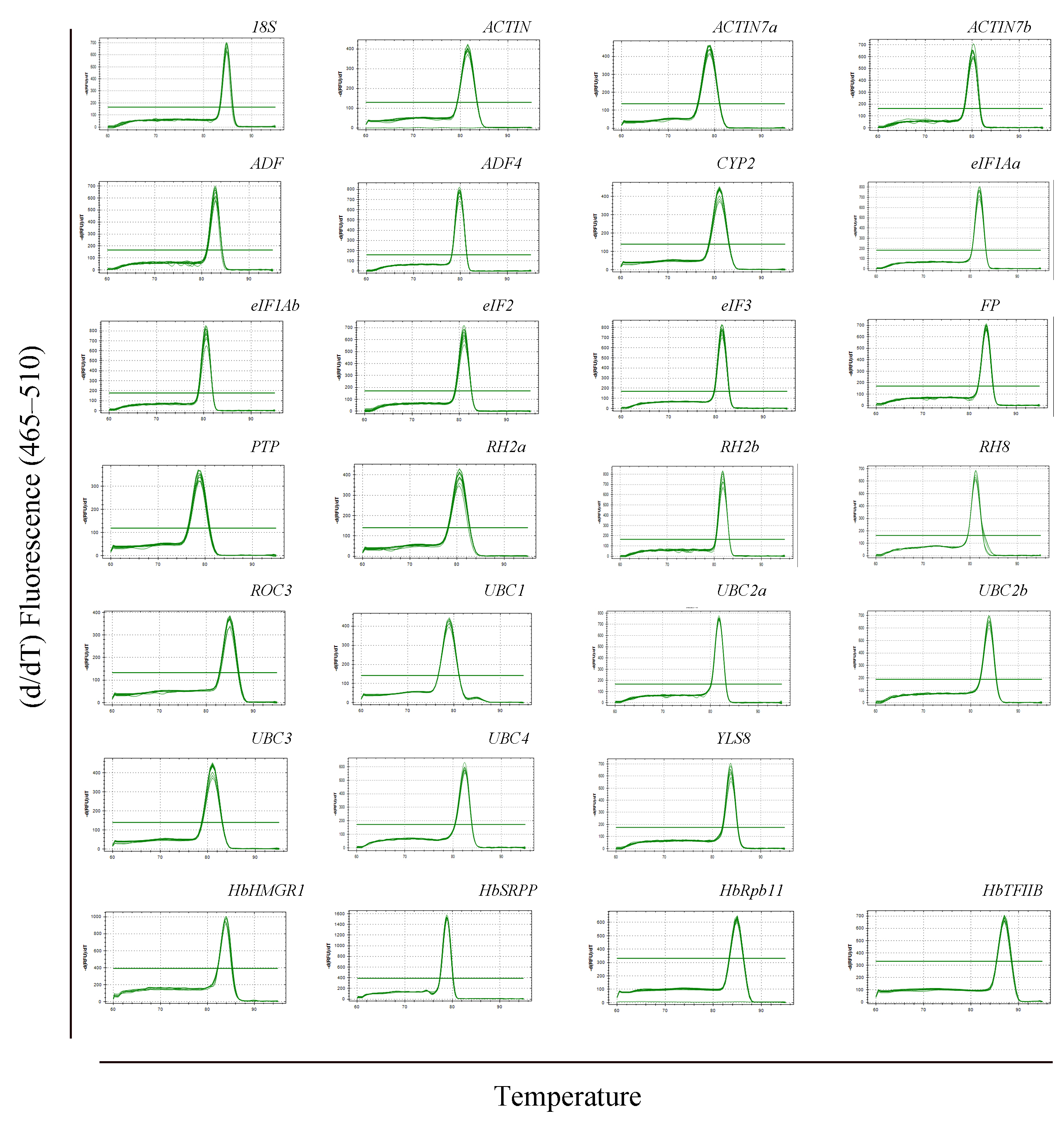

Supplement: FIGURE S1 — The melting curves of the 23 candidate reference genes and four latex regeneration-related genes. [file Image_1.JPEG]
